# Processing of Holographic Hydrogels in Liquid Media: A Study by High-Performance Liquid Chromatography and Diffraction Efficiency

**DOI:** 10.3390/polym14102089

**Published:** 2022-05-20

**Authors:** Kheloud Berramdane, Manuel G. Ramírez, Paola Zezza, María Isabel Lucío, María-José Bañuls, Ángel Maquieira, Marta Morales-Vidal, Augusto Beléndez, Inmaculada Pascual

**Affiliations:** 1 I.U. Física Aplicada a las Ciencias y las Tecnologías Universidad de Alicante, Carretera San Vicente del Raspeig s/n, 03690 San Vicente del Raspeig, Spain; bk35@alu.ua.es (K.B.); ramirez@ua.es (M.G.R.); marta.morales@ua.es (M.M.-V.); 2 Instituto Interuniversitario de Investigación de Reconocimiento Molecular y Desarrollo Tecnológico (IDM), Universitat Politècnica de València, Universitat de València, Camino de Vera s/n, 46022 Valencia, Spain; pzezza@doctor.upv.es (P.Z.); malube@upv.es (M.I.L.); mbpolo@upv.es (M.-J.B.); amaquieira@qim.upv.es (Á.M.); 3 Departamento de Química, Universitat Politècnica de València, Camino de Vera s/n, 46022 Valencia, Spain; 4 Departamento de Física, Ingeniería de Sistemas y Teoría de la Señal, Universidad de Alicante, Carretera San Vicente del Raspeig s/n, 03690 San Vicente del Raspeig, Spain; a.belendez@ua.es; 5 Departamento de Óptica, Farmacología y Anatomía, Universidad de Alicante, Carretera San Vicente del Raspeig s/n, 03690 San Vicente del Raspeig, Spain

**Keywords:** acrylamide-based hydrogel matrix, diffraction efficiency holographic volume grating, holographic transmission gratings, high-performance liquid chromatography, holographic sensing, temporal stability

## Abstract

The storage of time-stable holographic gratings in hydrogel matrices when the material is immersed in aqueous media is a real challenge at present. The optimization of the storage stages of the holograms must be properly investigated to identify the most suitable development processes. For this reason, this work is focused on the study of the optimization of the washing stages of the hydrogels based on acrylamide and *N*,*N*’-methylenebis(acrylamide) once unslanted transmission holograms have been stored. High-performance liquid chromatography and UV-visible measurements have been employed in our system to analyze the composition of the washing solutions. PBST and DMSO:H_2_O are used as solvents in the washing stages. The diffraction efficiencies are measured during the washing stages and after the storing of the holograms during several days in PBST. Maximum diffraction efficiencies of 38 and 27.6% are reached when PBST and DMSO:H_2_O are employed, respectively, for the washing process. Holograms show temporal stability after being stored immersed in PBST at 4 °C for 4 days.

## 1. Introduction

The design of sensors for qualitative and quantitative detection of different physical processes and chemical substances is very much needed in many fields such as agriculture, industry, environmental hazards, military, entertainment imaging, medical diagnostics, etc. In particular, holographic sensing is a research subject of great interest nowadays.

During the last decade, holography has acquired great importance since holographic devices can store information throughout the volume of the material [1]. A fundamental element of these devices is the recording material where the information is registered. Research groups around the world are concentrating all their efforts on obtaining a suitable material for this purpose [2]. It is important to obtain a material with a high refractive index modulation and high sensitivity as well as good optical quality. In addition, more recently, efforts have been made to find materials with high resolution [3]. Furthermore, in a hologram, any physical or chemical stimulus can produce changes in the diffraction grating spacing or in the refractive index modulation. This will result in a modification of the diffracted and transmitted beams that can be used to develop a detection method or a specific sensor [4,5,6]. Hologram based sensors were first proposed by Lowe’s group [7] and since then, they have been applied for the sensing of different analytes with great performance. 

Currently, holograms can be permanently recorded in various media like silver halide films, dichromate gelatin or photopolymers [8]. Typically, natural organic polymers or hydrogels are used as matrices, in which the holograms are stored [9]. 

Hydrogels are 3D polymeric networks with high swelling capacity in water and appropriate chemical, mechanical and biological features, which allow the storing of bioactive substances. Conventional hydrogels are prepared using synthetic polymers including polyacrylamide, poly(N-isopropylacrylamide) (PNIPAM), poly(vinyl alcohol), alkenes, alginates, synthetic or polysaccharide-based natural polymers, and several reaction types (covalent crosslinking, self-assembly, gelation) and activation modes (thermal, photochemical, and chemical) are employed for their synthesis [10,11]. The hydrogel matrices have a liquid permeable structure that allows the diffusion of molecules inside and are able to respond to external stimuli if they are properly engineered. Outstanding hydrogel features have been studied considerably in tissue engineering [12], drug release [13,14,15] and biosensors [16]. Hydrogels’ response is interesting as they can act as optical transducers for the quantitative detection of analytes in optical systems based on micro-lenses [17], holography [18,19,20,21,22], photonic crystals [23,24], or optical fibers [25,26,27] for glucose detection. In hydrogel based holographic sensors, periodic structures are embedded on the hydrogel matrices. The interaction of the analytes with the matrices modifies the index modulation and/or the periodicity of the grating. These modifications are reflected in changes in the intensity and the angle of the diffracted light by the stored grating, and so, the diffraction efficiency (*DE*), i.e., the ratio between the intensities of diffracted and incident beams [28]. This principle has been used for the sensing of pH [29], heavy metals [30], and ethanol [31] among others. 

Gratings in different formats have also been applied for the label-free detection of biomolecules [32,33]. Diffractive structures of bioreceptors have been patterned on the surface of optical waveguides and tailored to transduce the magnitude of biorecognition assays into the intensity of single peaks in the reflection spectrum [34]. In these approaches, the grating response changes after the biorecognition event takes place and it results in a modification of its diffraction efficiency. These biosensing systems are considered simple, inexpensive, and very efficient compared with other label-free sensing systems. This makes them especially interesting considering that, in 2020, optical biosensors were identified as the most lucrative technology segment [35].

Biosensing with hydrogel-based surface relief diffraction gratings (SRG) has been reported for the detection of Thrombin [36] and Human Immunoglobulin-G [37,38]. In addition, we have recently reported the detection of C-reactive proteins in buffer and certified blood serum using a SRG fabricated with a hydrogel functionalized with phosphorylcholine units [39]. However, to the best of our knowledge and despite the great performance of holographic sensing, no direct detection of biomolecules has been carried out with this technology. To do so, an appropriate smart design of the hydrogels to detect new analytes should be carried out and, in addition, the storing of the holograms inside these specific materials in aqueous media should be optimized.

Silver halide emulsions have been employed as photosensitive materials and they are utilized for the recording of silver-based amplitude holograms inside hydrogels [40] by the process employed in photography [41]. This implies long procedures and complicated fabrication processes. One of the most interesting photosensitive materials are photopolymers. Photopolymers are organic components polymerized by light. Their main advantage over silver halide emulsions in holography is that photopolymers are self-processing [42]. They do not need complicated chemical processes and stable holograms can be achieved with just simple UV exposure [3,43]. In addition, higher diffraction efficiencies and resolutions can be achieved as phase volume holograms can be obtained [44,45,46]. Moreover, the photopolymer components can be easily embedded in the matrix hydrogel [5,16] and these holograms can be used for sensing purposes [47,48]. 

Holographic biosensing needs the functionalization of the hydrogel with a biorecognition element. This step could be carried out before or after the holographic recording. As a first approach, we aim to investigate the holographic behavior of the hydrogel without the bioreceptor and the analysis of the holographic response in liquid media. Consequently, a systematic analysis of the storing processes of the holograms with photopolymers within hydrogels is still recommended to use them for sensitive biosensing purposes. Concretely, the aim of this work is to study the performance of the hologram during the successive washing steps that are necessary to eliminate the components that do not react during the recording process. In addition, the stability of these holograms is analyzed when the hydrogels are immersed in a solution of phosphate-buffered saline with Tween 20 (PBST), a commonly used medium for biosensing assays.

## 2. Materials and Methods

### 2.1. Material Preparation

Acrylamide (AAm), *N*,*N*’-methylenebis(acrylamide) (MBA), potassium persulfate (KPS), *N*,*N*,*N*’,*N*’-tetramethylethylenediamine (TEMED), triethanolamine (TEA), eosin yellowish (EY), dimethyl sulfoxide (DMSO), potassium phosphate dibasic, potassium phosphate monobasic, sodium chloride, potassium chloride and Tween-20 were purchased from Sigma-Aldrich Química SL (Madrid, Spain). PBST buffer 10 mM pH 7.4 consists of potassium phosphate dibasic 0.8 mM, potassium phosphate monobasic 2 mM, sodium chloride 137 mM, potassium chloride 2.7 mM and Tween-20 0.05% *v*/*v*. The preparation of the photopolymer material involved two stages. First, the hydrogel matrix was prepared. AAm (78.2 mg) and MBA (7.5 mg) were dissolved in 0.5 mL of distilled water. The solution was homogenized by stirring during 1 h. KPS (5.0 mg) was then added, and the solution was sonicated for 2 min until the KPS was completely dissolved. The solution was filtered using a 0.2 μm pore filter (Millipore, Burlington, Massachusetts, USA). Then, TEMED (1.2 µL) was added and mixed homogeneously by pipetting. 500 µL of the resulting solution were quickly deposited onto a levelled glass slide (7.5 cm × 2.5 cm) (Labbox Labware, S.L., SLIB-G10-050, Premia de Dalt, Spain) provided with a sticking mold (5.5 cm × 1.5 cm × 340 µm). The system was quickly sealed with another glass slide and tightened with two clamps. The hydrogel was allowed to polymerize at room temperature for 2 h. After the polymerization time, the hydrogel was removed from the mold, washed with distilled water and stored in water at 4 °C. The thickness of the hydrogel matrices was 340 ± 10 µm.

In the second stage, an incubator solution (IS) in DMSO:H_2_O (6:4 *v*/*v*) containing the next molar fraction was prepared: 0.0475 AAm (polymerizable monomer), 0.0121 MBA (crosslinker), 0.0030 TEA (co-initiator and plasticizer), 7.91·10^−6^ EY (photosensitizer dye), 0.2582 DMSO and 0.6791 H_2_O. Then, 700 µL of this solution were deposited over pieces of hydrogels with sizes of 2.6 × 1.5 cm for 20 min at 22 °C for imbibing the compounds within the hydrogel matrix. After this time, the hydrogel pieces (*n* ~ 1.43 at *λ* = 589 nm) were placed onto flat glass slides (*n* = 1.4699 at *λ* = 632.8 nm, SLIB-G10-050, Labbox) for the holographic recording. The process was carried out under controlled light conditions, to which the material was not sensitive.

### 2.2. Holographic Setup

**Figure 1:**
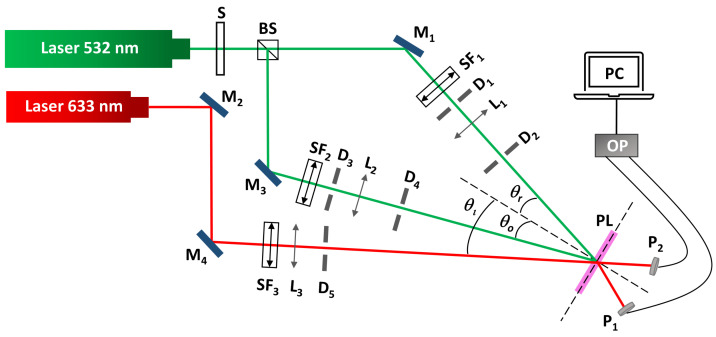
Holographic setup for transmission gratings. S: shutter; BS: beam splitter; SFi: spatial filters (microscope objective and pinhole); Mi: mirrors; Li: lens; Di: diaphragms; *θ*o and *θ*r: object and reference recording angle, *θ*i: incident reconstruction angle; PL: hydrogel matrix; Pi: photodetectors; OP: optical power meter; PC: data recorder.

Unslanted transmission volume phase holographic gratings were stored in the hydrogel matrices. The experimental setup used for the recording of the holographic gratings is shown in Figure 1. A continuous (CW) Nd:YVO_4_ laser (Verdi-2W, Coherent, Santa Clara, CA, USA) emitting at λ = 532 nm (at which the material is sensitive) was used. The laser beam was split into two secondary beams, object and reference beams, using a beam splitter (Newport, Irvine, CA, USA). The ratio of intensities between both beams was 1:1. Then, the beams were spatially filtered and collimated. The diameter of both beams was 0.35 cm. The holographic gratings were recorded by the appropriate interference between them. The object and reference beams were spatially overlapped at the sample with the recording angles *θ*o = *θ*r = *θ* = 18.7°, with respect to the normal incidence. The working total intensity (sum of both intensity beams measures in the hologram plane) was 19.3 ± 0.6 mW/cm^2^ and the exposure time was 8.0 ± 0.1 s. The laser beams have linear polarization perpendicular to the plane of incidence that allows optimal interference. According to Bragg’s law for symmetrical transmission geometry (Equation (1)), holograms were recorded at a theoretical spatial frequency of 1205 lines/mm (period *Λ* = 0.830 µm). The recording processes were carried out at 22 °C.

(1) $$\Lambda =\frac{\lambda }{2\sin\theta }$$

In the reconstruction stage, the diffracted and transmitted intensity were monitored in real-time with a He–Ne laser (Model 30995, REO, Boulder, CO, USA) positioned at Bragg’s angle (*θ*_i_ = *θ*_Bragg_ = 22.4° for *Λ* = 0.830 µm), at which the maximum diffraction efficiency (*DE*_max_) is obtained. This laser has linear polarization parallel to the plane of incidence. The holographic material is not sensitive at reconstructed wavelength of 632.8 nm. This Bragg angle must be shifted depending on the swelling of the hydrogels to obtain the angular scan.

### 2.3. Washing Stages

In order to remove the non-reacted compounds in the recording stage, several washing steps were carried out using two different solvents, DMSO:H_2_O 6:4 (*v*/*v*) and PBST. The hydrogel matrices with the stored holograms were repeatedly immersed for 5 min in 5 mL of these solvents. Washing solutions were recovered and stored for analysis with high-performance liquid chromatography (HPLC) and UV-visible analysis. The number of washing steps necessary for the complete removal of the non-reacted compounds is investigated in Section 3.1. The *DE* and angular shift of the unslanted transmission holograms were measured immediately after the recording and after several washing steps. After that, the hydrogels were immersed in PBST for 1 h and they were reconstructed again (Section 3.2). Finally, the hydrogels were immersed in PBST and stored at 4 °C in a fridge inside of a sealed container to preserve the material. Further stability studies were performed. The results are shown in Section 3.3.

### 2.4. High-pePrformance Liquid Chromatography (HPLC) and UV-Visible Analysis

An uHPLC 1260 Infinity Binary LC System (Agilent Technologies, Inc. Santa Clara, CA, USA) was used for the chromatographic studies of the washing solutions. Separation was performed on an Agilent zorbax eclipse XDB-C8 column (4.6 mm × 150 mm, 5 µm particle size). The column temperature was controlled at 30 °C. The mobile phase was 5.0% *v*/*v* acetonitrile in water with formic acid (0.1% *v*/*v*). The elution flow rate was 1.0 mL min^−1^. A diode array detector (DAD) at two wavelength, 210 and 248 nm, was used. The injection volume was 5 μL. The UV-visible analysis of the washing solutions was carried out in a double beam spectrophotometer ( Jasco, V-650, Madrid, Spain). 

## 3. Results

### 3.1. Study of the Composition of the Washing Solutions by HPLC and UV-Visible Analysis

The *DE*_max_ of the holograms stored in hydrogel and photopolymer matrices decreases over time due to the concentration gradient generated in the recording step. Molecular diffusion processes take place inside the material matrix. The generated polymer chains tend to diffuse towards non-exposed zones. Furthermore, the components that do not react in the non-exposed zones diffuse towards the exposed zones. These diffusion processes depend on the characteristics of the molecular components and the composition of the medium. A process to remove the concentration gradient is necessary to provide temporal stability to the stored holograms. Different techniques have been used with the aim to increase the stability of the holograms stored in photopolymeric materials such as exposure to ultraviolet light, dehydration of the photopolymer layers under controlled temperature conditions and incoherent light (LED lamp) [49,50,51]. LED lamp exposure post-recording is cheaper and simpler compared with other methods for photopolymer-based holograms. However, when these holograms are stored in stable hydrogel matrices in aqueous media, a method based on continuous washing of the post-recording material is more convenient. When an LED lamp is used, polymerization is produced in non-exposed zones. This polymerization is avoided when non-reacted components are removed by washing.

**Figure 2:**
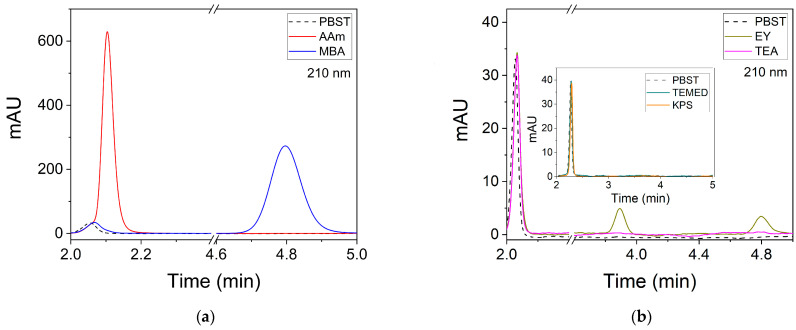
Chromatograms (mAU = mili OD at 210 nm) of standard solutions of the compounds used for the storage of holograms in the hydrogel matrices. (**a**) AAm and MBA; (**b**) EY, TEA, TEMED and KPS. The concentration of each compound is 50 ppm. The solvent used is PBST. Detection is carried out at 210 nm with a diode array detector.

In order to identify the number of washing steps that need to be performed to remove non-reacted components in the hologram recording stage, an HPLC study has been carried out with the washing solutions when PBST and DMSO:H_2_O 6:4 are used as solvents. For this, the chromatograms of standard solutions of all the compounds used for the preparation of the hydrogel matrix and the storage of the holograms are firstly obtained. The concentrations of these solutions are 50 ppm for all compounds in PBST. The chromatograms are shown in Figure 2. The resolution of the signals for AAm and MBA can be observed in Figure 2a. AAm is detected at a retention time of 2.10 min. This signal partially overlaps with the lowest intensity peak due to the PBST signal, which appears at a time of 2.06 min. However, this does not represent any inconvenience for the detection of AAm in the washing solutions. MBA presents a signal centered on a retention time of 4.80 min. Figure 2b shows the chromatogram for EY, TEA, TEMED and KPS. EY presents two bands with retention times of 3.89 and 4.80 min. The second signal coincides with the peak corresponding to MBA but with lower intensity. TEA, TEMED and KPS are not identified under the conditions of the chromatographic method used (Figure 2b and inset). It is necessary to point out that the PBST signal in Figure 2b and inset appears at slightly different retention times as the samples were analyzed on different dates. Small pressure oscillations in the system can slightly modify the retention times and the signal-to-noise ratio between different injections of the samples.

**Figure 3:**
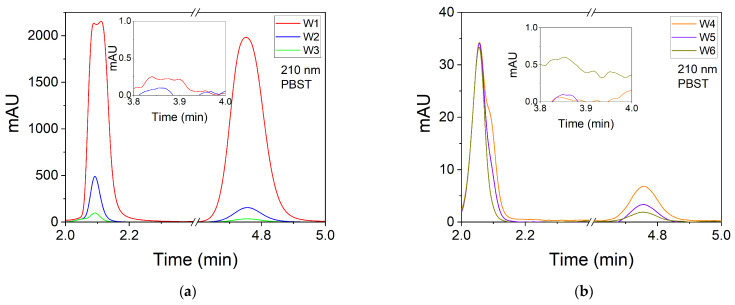

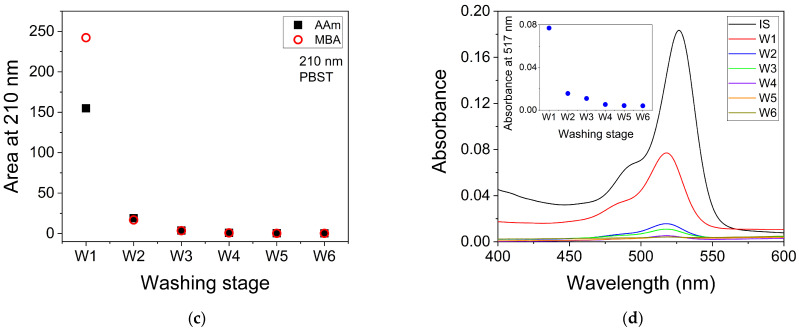
Chromatogram (mAU = mili OD at 210 nm) of washing solutions: (**a**) W1, W2 and W3; (**b**) W4, W5 and W6. Detection is carried out by absorption measurements at 210 nm; (**c**) Chromatogram area for AAm (black filled squares) and MBA (empty red circles) at 210 nm; (**d**) UV-Visible absorption spectra of incubator solution (IS) and washing solutions. Inset: Absorbance at 517 nm in the washing solutions. The solvent used is PBST.

Once the retention times of the different compounds used in this work have been identified, the chromatograms of the six washing solutions (W1 to W6) are obtained. The results are shown in Figure 3a,b. AAm is detected at a retention time of 2.09 min in the washing samples from W1 to W4. If the signals for W5 and W6 are compared with the PBST blank signal, it is confirmed that AAm is not detected in the last two washing stages (Figure 3b). MBA is detected at a retention time of 4.75 min in all wash samples. The signal for the sixth washing stage W6 is weak and could be confused with the noise signal. Therefore, it could not be confirmed with certainty that MBA is present in the last washing step. The areas of the chromatograms corresponding to the AAm and MBA signals show a decreasing exponential trend as a function of the washing steps (Figure 3c).

Under the method conditions used, EY is not detected in any of the wash samples. In aqueous solution, the EY absorption value at 210 nm is about half that at 517 nm where the maximum is found [52]. Due to the small concentrations of EY used, no signal is obtained in HPLC when a wavelength of 210 nm is used for its detection. In order to detect the presence of EY, UV-Visible analysis of the washing samples are carried out. The spectra of the samples and the incubator solution, in which the hydrogel is initially immersed, are shown in Figure 3d. EY is an anionic azo dye. Its spectrum in the incubator solution exhibits an intense band at 527 nm and a shoulder at 495 nm. When the first washing step with PBST is carried out (W1), the absorption maximum of EY is shifted at 517 nm (hypsochromic effect). Solvent polarity plays an important role in the spectral behavior of dyes. The position, intensity and shape of the absorption bands are influenced by the surrounding medium [53]. DMSO:H_2_O 6:4 is used as a solvent in the incubator solution while PBST is used in the hydrogel washing steps. Different scales based on spectroscopic measurements are used to measure the polarity of solvents [54] since the dielectric constant (ε) does not take into account all the solute–solvent interactions that may take place. However, as a simple approximation, the dielectric constant can be used to characterize the polarity of the medium. This parameter represents the ability of a certain substance to isolate charges. As the dielectric constant increases, the substance has a greater ability to stabilize charges and therefore a greater polarity. When the washing stages are carried out, the concentration of DMSO in the mixture decreases with respect to the concentration of water. Taking into account that the dielectric constant of water (*ε* = 80 at 20 °C) is greater than the value corresponding to DMSO (*ε* = 46.4 at 23 °C) [54], an increase in the dielectric constant of the medium is produced [55]. This increase justifies the hypsochromic shift when the first wash stage is performed. As the successive washing stages are performed, no changes in the position of the absorption maximum are produced while a clear decrease in the absorption intensity is observed. The EY signal is very weak from the fifth wash stage W5. The absorption at 517 nm as a function of the washing stage is shown in the inset of Figure 3d. As can be seen, the decrease of EY follows a decreasing exponential trend with respect to the successive washing steps. The largest amount of EY that has not reacted in the hologram formation stage is removed in the first two washing steps, W1 and W2. 

**Figure 4:**
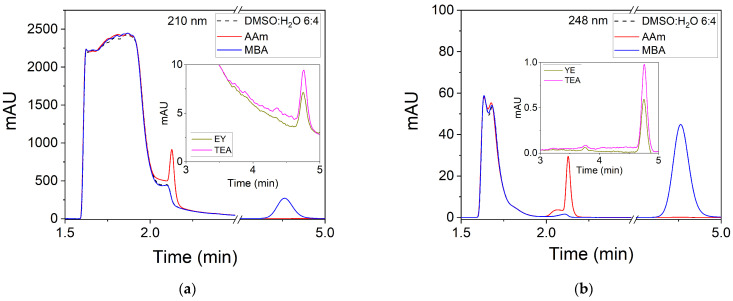
Chromatograms (mAU = mili OD at 210 and 248 nm) of standard solutions of AAm, MBA, EY and TEA. The concentration of each compound is 50 ppm. The solvent used is DMSO:H_2_O 6:4. Detection is carried out with a diode array detector at: (**a**) 210 nm and; (**b**) 248 nm.

Detection conditions must be changed when DMSO:H_2_O 6:4 is used as solvent for washing the hydrogels as DMSO exhibits an absorption band at 210 nm. The chromatograms of standard solutions with concentrations of 50 ppm of AAm and MBA are shown in Figure 4. When an absorption wavelength of 210 nm is used, the signal corresponding to AAm appears when the eluent front is ending (Figure 4a). This implies that the AAm peak overlaps with the signal from the DMSO:H_2_O 6:4 solvent and therefore its detection is difficult at low concentrations, i.e., when the concentrations of this compound in the washing solutions decrease. In order to solve this problem, an absorption wavelength of 248 nm was selected. Figure 4b illustrates that the AAm signal no longer overlaps with the solvent signal at 248 nm. AAm is detected at a retention time of 2.13 min for both absorption wavelengths. On the other hand, if the intensities of the AAm peaks are compared, there is a decrease from 925 mAU at 210 nm until 30 mAU at 248 nm. Despite this decrease in intensity, the 248 nm wavelength is used to detect AAm as there is no overlap with the solvent signal. The MBA signal is detected for both wavelengths at a retention time of 4.77 min without any interference from the solvent signal. The intensity of the signal is greater at 210 nm (272 mAU) compared to that obtained when a wavelength of 248 nm (45 mAU) is used for detection. The signals corresponding to EY and TEA are shown in the insets of Figure 4. These signals appear at the same retention time as MBA. Their intensities are around 4 mAU at 210 nm and less than 1 mAU at 248 nm. Considering that the intensity for the MBA signal is 282 mAU at 210 nm, the interference of EY and TEA does not represent a problem for the detection of MBA at this absorption wavelength.

**Figure 5:**
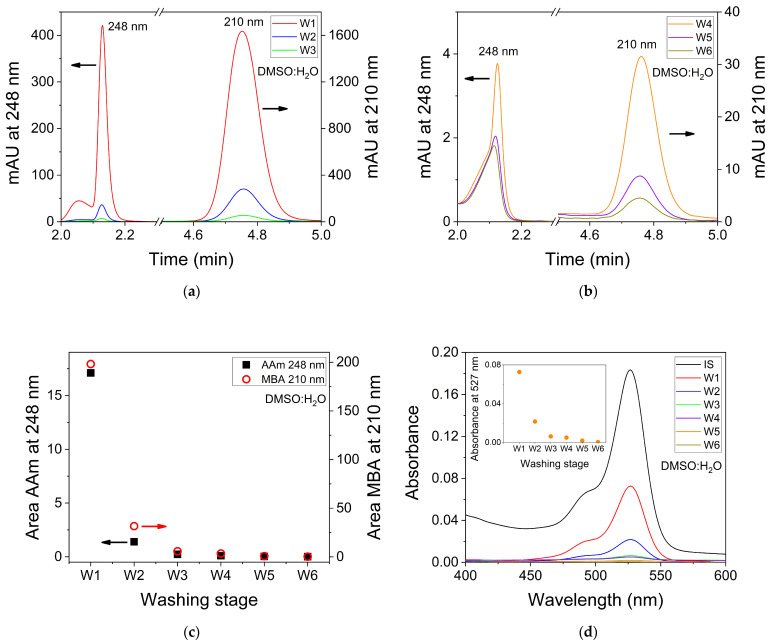
Chromatograms (mAU = mili OD at 210 and 248 nm) of washing solutions: (**a**) W1, W2 and W3; (**b**) W4, W5 and W6. Detection is carried out by absorption measurements at 248 nm for AAm and 210 nm for MBA; (**c**) HPLC area for AAm at 248 nm (black filled squares) and MBA at 210 nm (empty red circles); (**d**) UV-Vis absorption spectra of incubator solution and washing solutions. Inset: Absorbance at 527 nm of the washing solutions. The solvent used is DMSO:H_2_O 6:4.

Chromatograms of the six washing samples with DMSO:H_2_O 6:4 are shown in Figure 5a,b. In these figures, the left axis represents the absorption at 248 nm used for the detection of AAm and the right axis shows the absorption at 210 nm used for the detection of MBA in the wash solutions. AAm and MBA are detected at retention times of 2.13 and 4.76 min, respectively. The decrease in the intensity of the AAm and MBA peaks as the number of washing stages increases is clearly observed. For the last washing step W6, both compounds show signs of their presence. However, these signals are very weak and close to the detection limit. The areas of the chromatograms for AAm and MBA show the same decreasing exponential behavior compared to that observed when the solvent used is PBST (Figure 5c). Most of the non-reacted AAm and MBA amount are removed in the first (W1) and second wash steps (W2). The area of the AAm signals cannot be compared with those obtained when PBST is used as solvent since the absorption wavelengths used for their detection are different. On the other hand, the areas of the MBA signals when both PBST and DMSO:H_2_O 6:4 are employed can be compared since a wavelength of 210 nm has been used in both measurements. The area values for MBA are 242 and 198 when PBST and DMSO:H_2_O 6:4, respectively, are used for washing. More MBA is removed in the first wash step W1 when PBST is used. The difference in the value of the areas for W1 is small and may be due to different factors, such as different thicknesses of the hydrogel matrices and slight differences in the concentrations of MBA in the incubator solutions.

Following the same procedure for the EY detection in PBST, absorption spectra are measured for all the washing solutions in DMSO:H_2_O 6:4. These spectra and the one of the incubator solution are shown in Figure 5d. There is no change in the position of the wavelength of the absorption maximum since the solvent used for washing is the same as that used for the incubator solution (DMSO:H_2_O 6:4). A decrease in the absorption intensity is clearly observed. Concretely, an exponential decrease in the absorption is observed when the absorption maximum at 527 nm is monitored for each washing step (inset Figure 5d). The EY signal is close to zero from the fifth wash stage W5 onwards indicating that it is practically removed. 

Considering the results obtained by HPLC and UV-Visible spectroscopy, six washing steps are sufficient to remove the compounds that have not reacted in the transmission grating formation stage. In addition, three simultaneous washings with PBST are performed before definitively immersing the material in this solvent for subsequent storage.

### 3.2. Behaviour of the Diffraction Efficiency as a Function of the Washing Stages 

**Figure 6:**
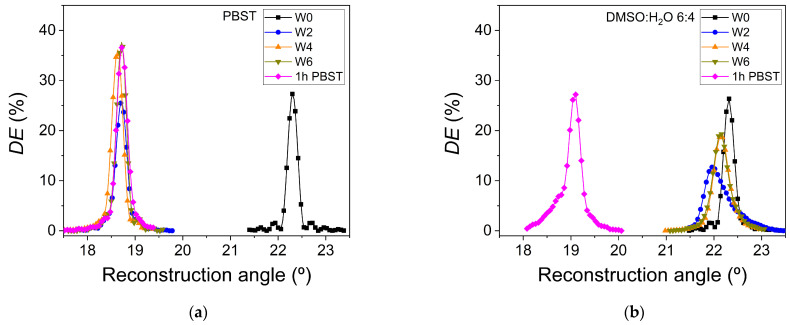
Diffraction efficiency as a function of the reconstruction angle for the two solvents used in the washing stage: (**a**) PBST; (**b**) DMSO:H_2_O 6:4. After exposure (W0, black square), after two washing steps (W2, blue circle), after four washing steps (W4, orange triangle), after six washing steps (W6, dark yellow triangle) and after one hour immersed in PBST (1 h PBST, magenta rhombus).

The angular scans of unslanted transmission gratings stored in the hydrogel matrices for the two solvents used in the washing stages are shown in Figure 6. To avoid repeated handling of the hydrogels, *DE* as a function of the reconstruction angle was measured immediately after the recording stage (W0, no washing) and after two, four, and six washing processes (W2, W4, and W6, respectively). Furthermore, the last angular scan was obtained after immersing the hologram in PBST for one hour at ambient conditions. The angular scans of the successive washing stages show asymmetries in the lateral lobes. These asymmetries are caused by the bending of the interference fringes inside of the holograms [56]. The maximum *DE* values and the reconstruction angle offset are influenced by bending. In Figure 6 it can be clearly observed that the asymmetries produced in the angular scans are more pronounced when DMSO:H_2_O 6:4 is used for washing. Therefore, from a point of view of obtaining a more defined holographic response, i.e., less bending, a PBST washing from the initial stage is more convenient. In addition, the different swelling of the hydrogel caused by the different polarity of the solvents used must be taken into account. The typical hydrogels based on AAm exhibit swelling in aqueous media and collapse in organic medium. This property can be used for their use as holographic sensors for such compounds. The equilibrium in the swelling of hydrogels depends on the balance between the cohesive forces that cause the solvent molecules to penetrate the hydrogel matrix and the dispersing forces acting on hydrated chains [57]. Other factors such as the degree of crosslinking of the AAm chains, pH, ionic strength, and temperature must be taken into account in the study of the swelling behavior of the hydrogels and their influence on the holographic parameters [58,59,60]. 

**Figure 7:**
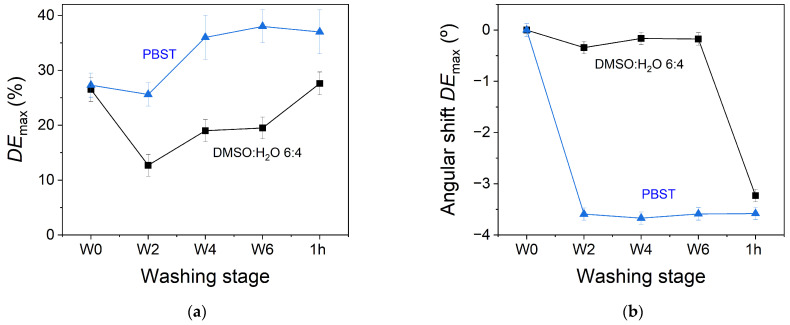
(**a**) Maximum diffraction efficiency (*DE*_max_) and (**b**) angular shifts with respect to the reconstruction angle of holographic hydrogels after the different for the washing stages with the two solvents used: DMSO:H_2_O 6:4 (black square) and PBST (blue triangle). W0 indicates no washing.

*DE*_max_ of the holographic hydrogels as function of the number of washing stages is shown in Figure 7a. When DMSO:H_2_O 6:4 is used, *DE*_max_ decreases from 26.5 in W0 until 12.7% after the second washing stage W2. This decrease may be due to excessive bending in the holographic fringes (Figure 6b). *DE*_max_ values for the fourth W4 and sixth W6 washing stages are both 19%. If PBST is employed, the *DE*_max_ drops slightly from 27.3% in W0 to 25.6% in the second washing stage W2 (Figure 7a). Taking the errors into account, it can be seen that *DE*_max_ does not practically change when the first two washing stages with PBST were carried out. An increase in *DE*_max_ takes place from the second W2 to the fourth W4 washing stage. From this stage, the value for *DE*_max_ practically remains constant until the last wash W6. In this washing stage, a *DE*_max_ value of 38% was obtained. Since the final aim is to keep the hydrogels immersed in PBST at a controlled pH, all the hydrogel matrices were immersed in this solvent for one hour to check their final holographic response. In the case of PBST, the *DE*_max_ has practically the same value as that obtained in the washing step W6. For DMSO:H_2_O 6:4, the *DE*_max_ value increased to 27.6%.

The angular shift with respect to the reconstruction angle for the holographic hydrogels after the washing stages is shown in Figure 7b, where W0 indicates no washing. A clear difference in the behavior of the angular shift is observed for the two solvents used. As expected, the smallest angular shift is observed when DMSO:H_2_0 6:4 is used. This solvent is the same as the one used for the incubation stage. Therefore, very small changes in the swelling of the hydrogel can be expected. The hydrogel based on AAm-MBA have hydrophilic character due to the presence of –CONH_2_ and –CONH– groups. The polarity of the solvent used for the washing stage influences the solvation of these groups and therefore the swelling. Since the dielectric constant for water is higher than for the DMSO:H_2_O 6:4 mixture, as discussed in Section 3.1, the angular shift is higher for PBST than for DMSO:H_2_O 6:4. This behavior follows the same trend as the swelling measurements found in AAm-based hydrogels when immersed in solvents with different polarity [58]. The angular shift with respect to the reconstruction angle for W0 grows as the swelling caused by the solvent increases. This increase in the swelling causes a broadening of the period of the hologram. According to Equation (1), the reconstruction angle, at which the maximum diffraction efficiency is obtained, is smaller as the period increases. As both holographic hydrogels are finally placed for 1 h in the same solvent, i.e., PBST, the final angular shifts are similar regardless of the solvent used for the washing.

### 3.3. Temporal Stability of the Transmission Gratings

**Figure 8:**
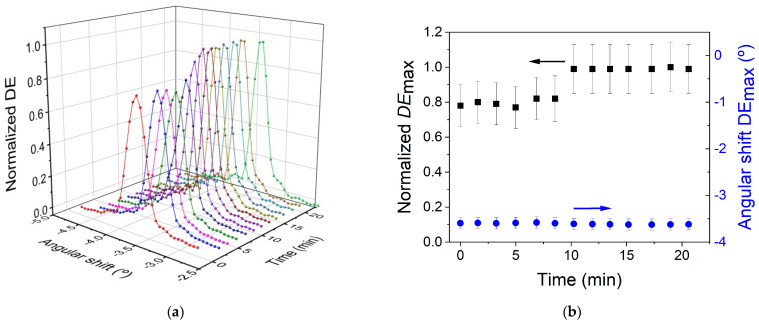
(**a**) Temporal evolution of the normalized *DE* and angular shift for holograms stored in hydrogel matrices out of the solvent during the reconstruction step. (**b**) Maximum values of the Normalized *DE*_max_ (black square) and angular shift of the *DE*_max_ (blue circle) as a function of time.

The temporal stability of the transmission gratings stored in the hydrogel matrices when PBST is used as the washing solvent has been obtained through two types of measurements. For the reconstruction step, the hydrogel matrix where the hologram is stored is placed on a glass slide out of the PBST solution, in which it was immersed and stored. The time that the hydrogel remains out of the solvent can affect to the holographic reading. Therefore first, the evolution of the *DE* and the angular shift with respect to the reconstruction angle for W0 was continuously measured as a function of the time that the holographic hydrogel was out of the solvent. These measurements were carried out at an ambient temperature and humidity of 23.8 °C and 53%, respectively. Figure 8a shows the angular scan as a function of time. From these measurements, the values of angular shift and normalized *DE*_max_ are obtained and plotted as a function of time (Figure 8b). Measurements are not performed for times greater than 20 min since the hydrogels do not remain stable on the glass support due to the exchange of water with the environment. Although the *DE*_max_ values remain stable within the error range of the measurements, two zones can be clearly observed. Normalized *DE*_max_ remains at a value of approximately 0.8 from the first measurement at time zero to the sixth measurement performed at a time of 8.6 min. From this time and until the end of measurements, normalized *DE*_max_ increases and remains stable at a value around 1.0. On the other hand, the angular shift of *DE*_max_ remains practically constant, taking the error into account, throughout the time interval in which the measurements are carried out. These results show that, under the temperature and humidity conditions used, the time elapsed between the manipulation of the hydrogel to place it on the glass support and the measurement of the angular response of the hologram is not a determining factor for the *DE* and angular shift determination.

**Figure 9:**
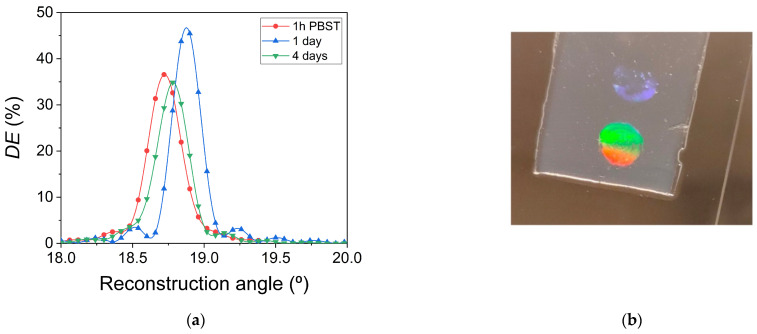
(**a**) *DE* as a function of the reconstruction angle for a transmission grating reconstructed immediately after being 1 h immersed in PBST solution at ambient conditions (red circles and line), 1 (blue tringles and line) and four days (green triangles and line) immersed in PBST solution and stored at 4 °C. (**b**) Image of the unslanted transmission gratings daylight illuminated.

The temporal stability of the transmission gratings immersed in PBST solution for several days has also been investigated. For this, reconstruction measurements were carried out at three different times. The results are shown in Figure 9a. The angular scan obtained when the hydrogel is immersed in PBST for 1 h has already been shown in Section 3.2. This measurement is used in Figure 9a for a correct study of the temporal stability. After this measurement, the transmission gratings are immersed in a PBST solution for 1 day at 4 °C. This temperature was selected in order to preserve the hydrogel material in better conditions. After this time, a *DE*_max_ value of 46.7% and angular shift of 0.2° are measured. The transmission grating was again immersed in a PBST solution and stored at the same temperature for three days. After this time, a *DE*_max_ value of 35.0% and angular shift less than 0.1° are measured. These results indicate that the holograms are time-stable when immersed in PBST for several days. However, the different *DE*_max_ values show that caution should be exercised when comparing reconstruction measurements performed at different times. A possible explanation for the different values of the *DE*_max_ can contemplate the temperature change, to which the material is subjected when the reconstruction measurement is carried out. More studies should be carried out with this type of hologram, stored in hydrogel matrices, to clarify these differences.

A transmission grating stored in a hydrogel matrix after four days immersed in a PBST solution is shown in Figure 9b. A second transmission grating can also be observed under the illumination angle of the image. The unexposed zones were practically transparent to daylight.

## 4. Conclusions

Unslanted transmission gratings with a spatial frequency of 1205 lines/mm have been stored in a hydrogel matrix based on AAm and MBA as crosslinker. A process of successive washing of the hydrogels with two solvents, PBST and DMSO:H_2_O 6:4, has been carried out to remove the components that have not reacted in the hologram generation stage. The number of washing stages required for the complete elimination of these compounds has been studied by means of HPLC and UV-Visible spectroscopy of the washing solutions when the dimensions of the hydrogel are fixed. Considering the results obtained, we can conclude that six washing stages are required to remove the unreacted compounds for both solvents in our system. The physico-chemical characteristics of the solvents used in the washing stage influence the holographic responses of the material. When DMSO:H_2_O 6:4 is used, a greater bending is obtained in the angular scan measurements. The *DE*_max_ obtained at the end of the washing process with PBST is greater compared to that obtained when DMSO:H_2_O 6:4 is used. The temporal stability of the PBST washed transmission gratings has also been measured. The results indicate that the *DE*_max_ and angular shift values are time-stable when measured under controlled environmental conditions for 20 min. Holograms also show temporal stability when stored immersed in PBST at 4 °C for several days. This study shed light on the storing of holograms in hydrogel matrixes that should be immersed in solvents to preserve their stability. In addition, these holograms and procedures can be useful for the design of holography biosensors as PBST is one of the most common solvents used in the detection of biomolecules. Since the maximum values of the diffraction efficiencies and the reconstruction angle shift are influenced by bending, this parameter will be studied in more detail in future works. On the other hand, the different response observed in the holograms when they are immersed in PBST and DMSO:H_2_O opens the possibility of their use for the sensing of organic solvents. This will be also explored in future studies.

## Data Availability

The data presented in this study are available on request from the corresponding author.
